# Complexities in comparing the impact of costimulatory domains on approved CD19 CAR functionality

**DOI:** 10.1186/s12967-023-04372-4

**Published:** 2023-07-30

**Authors:** Richard Smith, Rhine Shen

**Affiliations:** 1 Kite Pharma Inc, Emeryville, CA 94080 USA; 2 Kite Pharma Inc, Santa Monica, CA 90404 USA

**Keywords:** CAR-T, Costimulation, CD28, 4-1BB, CD19

## Abstract

Chimeric antigen receptors (CARs) are engineered to target T cells specifically to tumor cells, resulting in the engineered T cell killing the tumor cell. This technology has been developed to target a range of cancers, with the most notable successes in the treatment of B-cell malignancies where four approved therapies, all targeting CD19, are on the market. These four products differ in the costimulation domains, with axicabtagene ciloleucel (Yescarta) and brexucabtagene autoleucel (Tecartus) both utilizing the CD28 costimulation domain whilst tisagenlecleucel (Kymriah) and lisocabtagene maraleucel (Breyanzi) both utilizing the 4-1BB costimulation domain. There are clearly defined differences in how the CD28 and 4-1BB domains signal, yet it is difficult to ascertain which domain affords a superior mechanism of action given many other differences between these products, including overall CAR architecture and manufacturing methods. Additionally, while in vitro and preclinical in vivo studies have compared CARs with different costimulation domains, it remains a challenge to extrapolate differences observed in this biology across different experimental systems to the overall product performance. While there has been extensive preclinical and clinical work looking at CARs with a variety of targeting domains and architectures, this review will focus on the differences between the four marketed anti-CD19 CAR-Ts, with an additional focus on the impact of hinge and transmembrane domain on CAR activity and interaction with the target cell as well as other proteins on the surface of the T-cell.

## Background

**Fig. 1:**
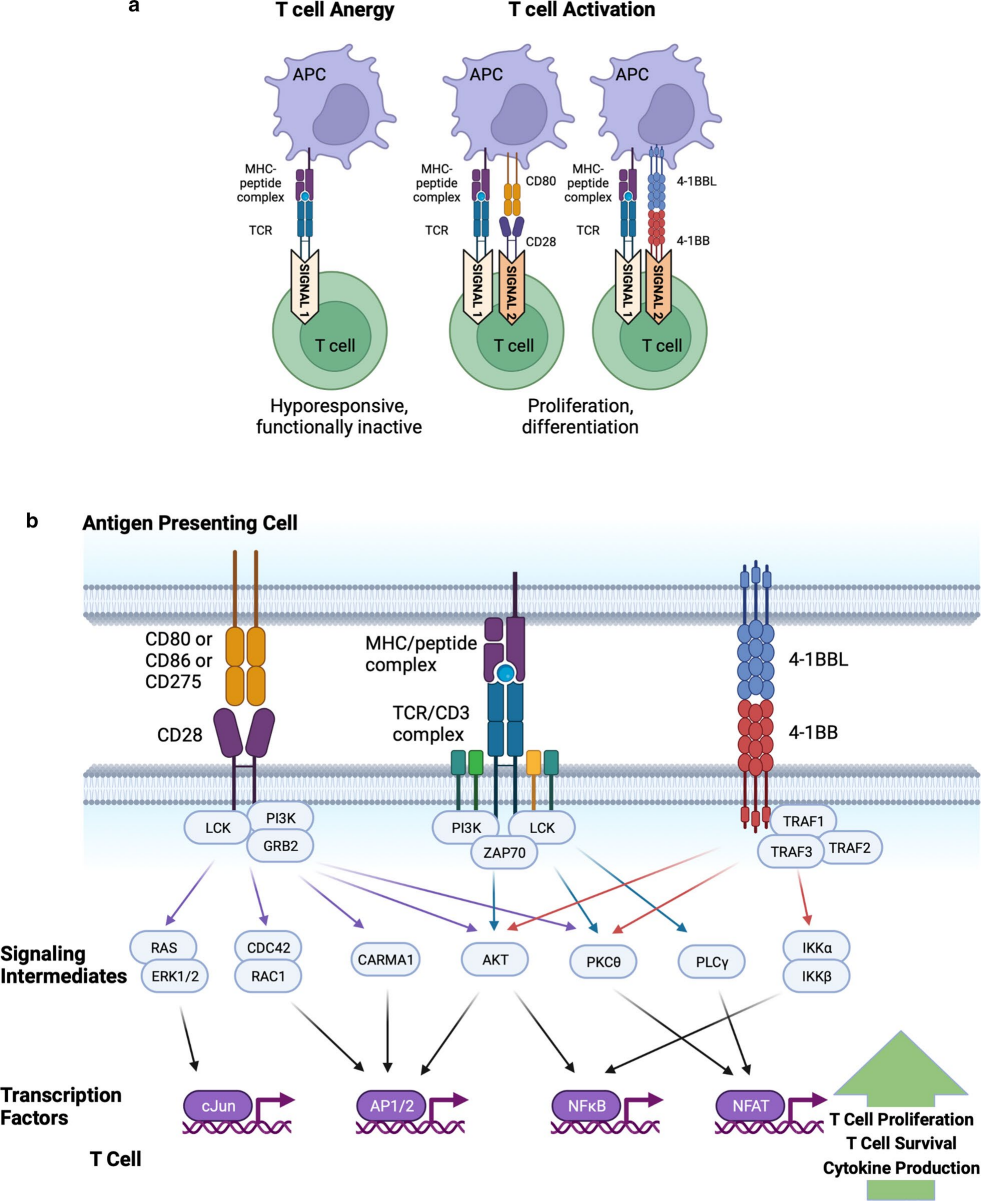
**a** The involvement of native CD28 and 4-1BB costimulation in T cell activation. T cell activation requires two signals. The first is provided by recognition of a specific peptide antigen held by MHC class II on the surface of an antigen presenting cell (APC) by the T cell receptor (TCR) complex. Professional APCs also provide a second signal through engagement of costimulatory molecules on the surface of the T cell; this second signal is essential for survival and expansion of the T cell. This figure highlights two costimulators; CD28, which is recognized by APC surface proteins CD80 and CD86 and 4-1BB, which is recognized by APC surface protein 4-1BBL. Created with BioRender.com. **b** A simplified representation of the integration of TCR signaling with costimulatory signals. The primary contact between the T cell and the APC is via the TCR/CD3 complex on the T cell and the MHC/peptide complex on the APC, initiating ‘signal 1’. CD28 and 4-1BB signaling (‘signal 2’) is then triggered by engagement with their ligands on the surface of the APC. This triggers dimerization of CD28, or trimerization of 4-1BB, resulting in recruitment of a variety of signaling molecules on the cytosolic side of the T cell membrane. It is important to note that the downstream signals from the TCR/CD3 complex overlap with those from CD28 and 4-1BB, notably via the PKCθ and AKT pathways. This in turn ultimately leads to transcription and translation of genes required to drive cytokine production (particularly IL2 and IFNγ), T cell proliferation and anti-apoptotic signals. Created with BioRender.com

Chimeric antigen receptors (CARs) have shown exceptional promise in the clinic in the treatment of hematological malignancies, introducing engineered receptors into a patient’s own T cells to target cancer cells. CARs are designed with an extracellular domain that binds to a tumor cell-surface antigen and cytosolic domains that drive T-cell activation, with the goal of trying to recapitulate the process of T cell activation in response to a foreign antigen, while redirecting the T cell to killing a tumor cell. An effective T-cell response to a target antigen requires two signals [1]. The first comes from engagement of the T-cell receptor (TCR) complex with the MHC-peptide complex on the antigen presenting cell. This signal provides recognition to a specific antigen. However, this signal alone will induce T-cell anergy, a critical mechanism for establishing tolerance to self-antigens [2]. Professional antigen presenting cells are able to provide a second signal via engagement with costimulatory receptors such as CD28 [3] and 4-1BB [4] on the surface of the T-cell, thus initiating a response to the antigen being presented by the MHC (Fig. 1a). CD28 and 4-1BB do initiate different signaling pathways (Fig. 1b), though with significant overlap with the downstream signals from the TCR complex. The overall outcomes (IL-2 production, T cell proliferation and anti-apoptotic signals) are broadly similar [5].

**Fig. 2:**
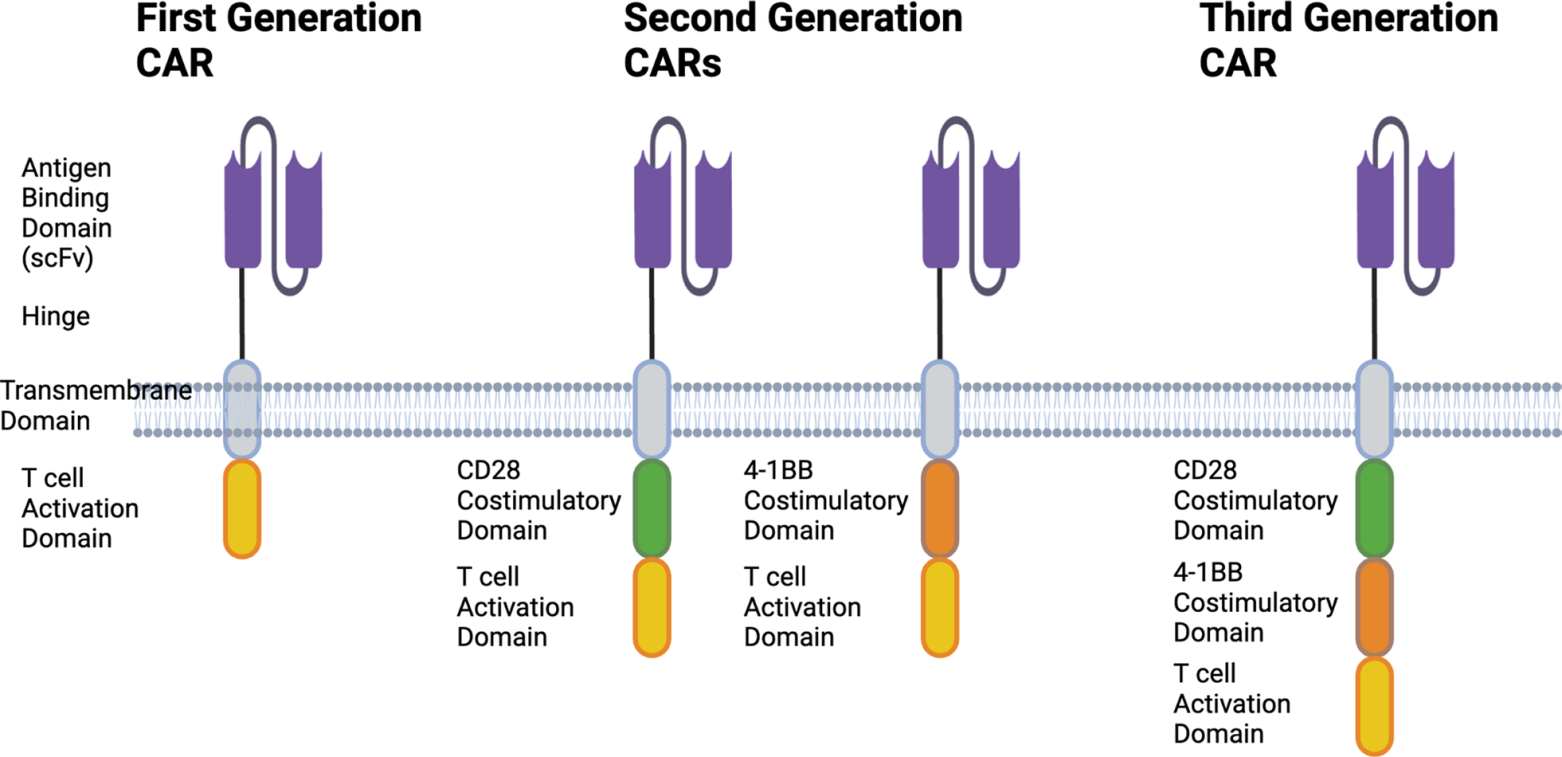
First, second and third generation CARs. First generation CARs consist of an antigen-binding scFv fused via a hinge to the transmembrane and activation domain from CD3ζ. Second generation CARs use a variety of hinges and transmembrane domains, plus a single costimulatory domain in addition to the antigen-binding scFv and CD3ζ activation domain. This enables them to recapitulate the two signals required for full T-cell activation. Third generation CARs build on the basic structure of the second generation CAR by adding a second costimulation domain. Created with BioRender.com

First generation CARs consisted of extracellular domains including a tumor-antigen specific single chain antibody fragment (scFv) linked to the CD3ζ signaling domain [6, 7]. While first-generation CARs induced an antigen-specific signal, anti-tumor activity in preclinical models was limited. Inclusion of a costimulation domain in second generation CARs provided the extra signal required to fully activate the T cell and drive an anti-tumor response [8], and clearly demonstrated superiority over first generation CARs in rodent models [9–12] (Fig. 2). Third generation CARs attempted to further enhance the costimulatory signal by stacking two or more costimulatory domains into a single CAR [13]. There was nonetheless mixed success with third generation CARs (reviewed in [14]), possibly underscored by the complex spatial requirements for effective engagement of the tandem cytosolic costimulatory domains with multiple effectors [15]. Several different costimulatory domains, including OX40, CD2 and CD27 have been studied [8], however the most common domains used in the field are CD28 and 4-1BB. The four marketed anti-CD19 CARs use either CD28 or 4-1BB costimulatory domains in a second-generation format.

### Comparing the four approved anti-CD19 CAR-T products

The architecture and manufacturing processes used for the four approved anti-CD19 CAR-T therapies are summarized in Table 1. All four CARs utilize the same binder, an scFv derived from a murine anti-human CD19 antibody known as FMC63 [16], and the same activation domain, CD3ζ [13]. The rest of the architecture is significantly different. Axi-cel [17–20] and brexu-cel [21–23] use the same CAR, which has hinge, transmembrane and costimulation domains derived from CD28. Tisa-cel [24–26] uses hinge and transmembrane domains derived from CD8α, and the 4-1BB costimulation domain. Liso-cel [27, 28] uses a hinge derived from IgG4, the CD28 transmembrane domain and the 4-1BB costimulation domain.

**Table 1:** A summary of the architectures and manufacturing processes for the four approved anti-CD19 CARs, axicabtagene ciloleucel (axi-cel), brexucabtagene autoleucel (brexu-cel), tisagenlecleucel (tisa-cel) and lisocabtagene maraleucel (liso-cel)

	Axi-Cel	Brexu-Cel	Tisa-Cel	Liso-Cel
*Architecture*				
Anti-CD19 scFv	FMC63	FMC63	FMC63	FMC63
Hinge	CD28	CD28	CD8α	IgG4
Transmembrane domain	CD28	CD28	CD8α	CD28
Costimulatory domain	CD28	CD28	4-1BB	4-1BB
Activation domain	CD3ζ	CD3ζ	CD3ζ	CD3ζ
*Manufacturing*				
Viral vector	γ-Retrovirus	γ-Retrovirus	Lentivirus	Lentivirus
Promoter	MSCV	MSCV	EF1α	EF1α
Starting material	PBMCs	CD3 + enriched	CD3 + enriched	Separated CD4 and CD8
Activation	Anti-CD3 coated bag + IL2	Anti-CD3 and anti-CD28 coated bag + IL2	Expansion with beads coated with anti-CD3 and anti-CD28	Independent activation of CD4 and CD8 with beads coated with anti-CD3 and anti-CD28
CD4:CD8 in final product	Patient dependent	Patient dependent	Patient dependent	Defined CD4:CD8

The native CD28 and 4-1BB receptors differ in the downstream signaling cascades, predominantly at the level of distinct protein binding partners recruited to the cytosolic domains following receptor-ligand engagement (Fig. 1b). However, analyses of activation and phosphorylation events following stimulation reveal few, if any, converged signaling differences [29]. Even an unbiased mass spectroscopic phosphoproteomic analysis looking at over 26,000 phosphorylation events across 4849 proteins revealed that a majority of phosphorylation events were shared between both costimulation domains (when the rest of the CAR architecture was held constant) [5]. The key difference was instead in the magnitude and kinetics of the signals, with the CD28-based anti-CD19 CAR inducing a significantly faster signal with greater amplitude. The CD28 CAR also exhibited a low level of basal CD28 phosphorylation that correlated with increased levels of Lck associated with the CAR, which was not seen in the 4-1BB-based anti-CD19 CAR. While this did correlate with potent cytotoxicity in vitro, the CD28-based CARs use in this study were not as effective at controlling a tumor in a rodent model as their 4-1BB-based counterpart, with the former cells becoming more rapidly exhausted and failing to persist. The authors do note the limitations of their analyses, with the in vitro activation being performed with antigen coated beads, thus excluding the contribution of other costimulators that would be found on tumor cells. Also, they only looked at one architecture (FMC63 anti-CD19 scFv plus IgG4 hinge plus CD28 transmembrane domain). A recent study indicated differences in T-cell dysfunction arising from chronic in vitro stimulation via a CAR with a CD28 costimulation domain, which exhibited a classical T-cell exhaustion profile, versus a CAR with a 4-1BB costimulation domain which exhibited a novel FOXO3-mediated dysfunction profile [30]. The authors showed a similar transcriptional profile in Tisa-cel CAR-T cells taken from a single patient who failed to respond. Further studies are needed to validate this observation in additional patients, and to understand if this novel profile is seen in other CAR-T cells that utilize a 4-1BB costimulation domain.

### Functional impact of the hinge and transmembrane domains within CD19-directed CARs

While there has been much focus on the impact of CD28 versus 4-1BB costimulation domains on CAR activity, the choice of hinge and transmembrane domain can have a profound impact [31]. This is evident from the range of results seen in preclinical rodent studies that have directly compared different costimulatory domains in anti-CD19 CARs, with some studies indicating CD28 costimulation yields a better response [32–36], while others indicate a better response when a 4-1BB costimulation domain is used [12, 37, 38]. It is important to note that it is impossible to draw clear conclusions from these studies as to which costimulatory domain in general is better (CD28 or 4-1BB) given the additional differences (hinge and transmembrane domains) in architectures used, as well as the different model systems in which they were tested. If we start to look at the various components of CAR architecture at a molecular level, we can begin to determine their relative contributions, and how they interact with each other, in defining the properties of a given CAR.

The approved CD19 CARs cover the range of widely used hinges, utilizing immunoglobin (Ig) family derived domains. Ig domains are found in over 750 extracellular proteins, not just immunoglobulins (or antibodies), and consist of around 125 amino acids forming an approximately globular structure. They are frequently found in membrane proteins where they form stable modular components of the extracellular domain. These favorable properties have supported their use in CAR hinge domains. These domains provide both structure and function. Ig domains from IgG1 antibodies mediate engagement with Fc receptors on myeloid cells, a property that has negated their use as CAR hinges [39, 40]. Other classes of antibody, such as IgG4, do not bind to cell surface receptors and have been used successfully as CAR hinges as in the case of liso-cel [40]. Another alternative is to use immunoglobulin-like domains from non-antibody proteins such as CD28 (axi-cel and brexu-cel) or CD8α (tisa-cel). Initially these sequences were considered to be largely inert, beyond providing appropriate presentation of the scFv to its cognate antigen on the target cell. However, there is an increasing body of evidence pointing towards the choice of hinge and transmembrane domain greatly impacting CAR activity [41–44].

A comparison of CD28 and CD8α-derived hinge and transmembrane domains with an anti-VEGFR2 scFv was performed by Fujiwara et al., showing that first-generation CARs with CD28-derived sequences had greater cytotoxic and cytokine-producing activity than equivalent CARs with CD8α derived hinge and transmembrane sequences in mouse CAR-T cells [41]. They then tested second-generation CARs using CD28 costimulation domains, and either CD28- or CD8α-derived hinge and transmembrane domains in human CAR-T cells. In this case the CARs with CD28-derived hinge and transmembrane domain had higher cytotoxicity activity, when controlling for CAR expression [41]. These data suggest that axi-cel may mediate pronounced cytotoxicity relative to liso-cel and tisa-cel, which do not harbor a CD28 hinge.

What could be the mechanism driving this difference? Further work from the same group has indicated that differences in glycosylation and the propensity for intermolecular disulfide bridging can influence antigen-dependent and independent activity [45]. This should not be surprising as native CD28 and 4-1BB receptors require multimerization (induced by engagement with their ligands) to be activated. In fact, first generation CARs require inclusion of CD3ζ transmembrane elements for activity, presumably enabling CAR dimerization and incorporation of the CAR into the immune synapse [46]. Dimerization of endogenous CD28 following engagement with its ligands, CD80 and CD86, results in recruitment of signaling molecules such as PI3 kinase, Lck and GRB2/GRAP2 to the cytosolic costimulatory domain, driving the second signal required for T cell activation [3] (Fig. 1b). The costimulatory domain in a CAR generates an equivalent signal when the CAR engages with an antigen on a tumor cell. It has been demonstrated that CD28 hinge/ transmembrane domain CARs can heterodimerize with native CD28 on the T cell surface [47]. Mueller et al. compared CARs consisting of FMC63 scFv and 4-1BB costimulatory domain (chosen to avoid any potential CD28 CAR cytoplasmic domain-driven association with native CD28) with combinations of IgG4, CD28 and CD8α hinges and CD28 and CD8α transmembrane domains. These CARs were introduced into T cells by CRISPR gene editing to better control equivalent levels of expression. Formation of CAR-CD28 heterodimers reduced the amount of free CD28 monomers on the T cell surface. Additionally, the heterodimers were unresponsive to the native CD28 ligands CD80 and CD86 (which is reassuring as this was viewed as a potential route to CD19-independent CAR activation). The recruitment of native CD28 to the CAR, and thus the immune synapse, may explain the stronger signal and increased sensitivity to lower antigen levels when using the CD28 hinge and transmembrane domains. A similar pairing, but to a lesser degree was seen with the CD8α hinge and transmembrane domain, while it was completely absent with the IgG4 hinge. Three-dimensional modeling of the structures of the various ECDs does suggest a structural basis for preference of CD28 hinge and transmembrane domain > CD8α hinge and transmembrane domain > IgG4 hinge, with potential stabilization of the native CD28 - CAR hinge-TM domain heterodimer mediated through access to an unpaired cysteine in the extracellular domain of native CD28 by an equivalent residue in either the CD28 hinge and transmembrane domain or the CD8α hinge and transmembrane domain. The IgG4 hinge lacks a suitable free cysteine in the interface region. In contrast the modeling shows how the three different CAR hinges can homodimerize following engagement of their associated binding domains with their cognate targets.

Majzner et al. have investigated how these differences manifest at a mechanistic level [48]. Utilizing confocal microscopy, they showed that FMC63-derived CARs had similar distribution on the T cell surface, irrespective of the hinge used, but that CD28 hinge/ transmembrane domains induced faster synapse formation than CARs incorporating a CD8α hinge/ transmembrane domain. This was assessed by measuring recruitment of a ZAP70-red fluorescent protein fusion to the intracellular side of the synapse. This is not to say that choice of costimulation domain does not impact activity; in this study it was shown that T cell activation kinetics (with FMC63 as a binder with CD28 hinge and transmembrane domains) were faster with the CD28 costimulatory domain than 4-1BB. Additionally, CD28 hinge and transmembrane domains conferred greater sensitivity to lower antigen levels than the CD8α hinge and transmembrane domains, suggesting higher potency in conditions where target expression may be lower. This has clinical impact that when targeting a population of tumor cells with a range of target antigen expression even cells expressing low levels of antigen will be eliminated [49]. It is notable that using either the 4-1BB or CD28 costimulatory domain in the context of the CD28 hinge and transmembrane domain resulted in CAR-Ts that had equivalent anti-tumor activity in an in vivo model of a CD19-low expressing leukemia. For CD19, where on target/ off tumor cytotoxicity results in the clinically manageable outcome of B cell aplasia, this could provide a significant advantage in driving a complete response. However, many solid tumor targeting concepts rely on differential expression of antigen between healthy tissue and tumor for establishing a therapeutic window [50–52].

Expression of the CAR on the cell surface is dependent on the CAR protein folding into its correct three-dimensional shape as it is produced inside the cell and then being transported to the cell surface. While the choice of hinge and transmembrane domain can influence how efficiently the CAR folds, the choice of scFv can have a more profound impact on CAR folding. Not all antibodies can be successfully converted into an scFv, resulting in mis-alignment between the heavy and light chain variable regions that can reveal hydrophobic ‘sticky patches’ that can cause the CAR to aggregate within the cell and not reach the surface [53–56]. In instances when a sub-optimally folded CAR gets to the cell surface this can result in ‘tonic signaling’ or signaling independent of antigen engagement as a result of CAR aggregation [57–59]. As all approved anti-CD19 CAR-Ts use the same scFv this can be excluded as a variable in impacting CAR expression or tonic signaling.

### Clinical data comparison of 41BB vs. CD28 CARs

As already noted with preclinical studies, it is difficult to infer patterns driven solely by choice of costimulatory domain across clinical studies for a number of reasons [60]. We have described how the rest of the CAR architecture has a strong influence on overall activity, even if the same scFv (as is the case with the four marketed anti-CD19 CAR-T therapies) is used. The method of cell manufacturing (summarized in Table 1) [61], including choice of vector, pre-transduction processing of apheresed material, activation method and length of culture can have a profound impact on the final product in terms of phenotype and overall potency. Patient baseline characteristics are critical, from the type and burden of disease, prior treatments, use of bridging chemotherapy, the lymphodepletion regimen used [17, 24, 62–64] as well as CAR T dose. Finally, there is a lack of consistency in how durable responses are assessed, including follow-up which may be complicated by the patient receiving consolidative allogenic hematopoietic stem cell transplantation. In addition, a range of different scales for grading adverse events are used [65–67]. A detailed summary reviewing clinical data across multiple trials for the four marketed anti-CD19 CAR-T therapies has recently been published by Cappell and Kochenderfer [60]. In general overall complete response (CR) rates are comparable; indeed a real-world retrospective analysis of B cell non-Hodgkins lymphoma patients who received either axi-cel or tisa-cel reported similar CR rates with both products [68].

Despite the challenges in comparing both responses and adverse events, Davey et al. [69] performed an analysis of a number of trials looking at different anti-CD19 CARs [17, 24, 62, 63, 70–84]. In this analysis of published trial data, they calculated CR rate as the number of patients who achieved CR at any point post-infusion expressed as a percentage of the total patients in the trial. Overall, FMC63-CD28-CD28-CD28-3ζ CARs have a slightly increased CR rate relative to FMC63-CD8α-CD8α-41BB-3ζ CARs in the studies included in this analysis (60% versus 50%). Interestingly a single trial with a FMC63-CD28-CD28-41BB-3ζ CAR had a very high CR rate (80%), but this only covered 10 patients [73]. In terms of CRS, there is a higher incidence at grades 1 and 2 with CARs using the CD28 hinge and transmembrane domains (73% versus 39% for CARs using other architectures), though there is insufficient data to determine if this differs based on the costimulatory domain used, given that only 10 patients in a single trial received a FMC63-CD28-CD28-41BB-3ζ CAR, versus 501 patients with published data who received FMC63-CD28-CD28-CD28-3ζ CAR. Interestingly the rate of CRS at grades 3 and higher is similar across all architectures (approximately 13%). A similar pattern can be seen for neurotoxicity, though as the authors note there are significant differences in the methods used for defining neurotoxicity events, particularly at low grade [85]. That being said, there is a clear trend in this analysis towards severe neurotoxicity events in patients treated with CARs utilizing the CD28 hinge and transmembrane domains. In addition, onset of CRS is more rapid with CARs containing a CD28 costimulatory domain (1–3 days) [17, 63] versus CARs containing 4-1BB (3–5 days) [62, 64]. It should be noted however that incidence and severity of both CRS and neurotoxicity can also be influenced by disease burden, strength and method of lymphodepletion and CAR-T dose (reviewed in [86]).

Cellular kinetics of axi-cel and tisa-cel are well characterized in B-ALL and LBCL and generally exhibit rapid expansion within 2 weeks post-infusion followed by multiexponential decline. For tisa-cel and liso-cel, peak expansion occurs at 12 days, and persists in the peripheral blood for up to 2 years [25, 64]. In comparison, axi-cel expands more robustly upfront to a higher peak at 7 days in patients with DLBCL [17]. While peak levels of tisa-cel are considerably lower than axi-cel, dosing is up to 3-fold higher [62, 87]. Across products, cell expansion strongly correlates with objective response that is further impacted by tumor burden [64, 87, 88]. Persistence is considered a contributor to overall efficacy of CAR-T products. Multiple reports of persisting CAR-Ts with 4-1BB costimulatory domains in patients with ongoing CRs up to 2 years post-infusion are reported [24, 62, 64, 72, 76, 89], while many early trials reporting limited persistence of CAR-Ts with CD28 costimulatory domains [18, 19, 71, 83]. Consistently, axi-cel does not persist as readily as liso-cel and tisacel, and while some axi-cel patients maintain detectable low levels of CAR, persistence in the periphery is not required for durable response for axi-cel. Of course, these differences can also be influenced by the same range of variables beyond the costimulation domain previously discussed. In particular, axi-cel and brexu-cel are delivered by a gamma retrovirus that can only infect proliferating cells, while tisa-cel and liso-cel are delivered by lentivirus, which can infect non-proliferating cells that may be in a less differentiated state and have a greater potential to survive. That being said, in one trial 51% of patients treated with axi-cel showed complete remissions lasting over three years, with remissions of over 9 years still ongoing [90]. This is also borne out by data from the ZUMA-1 trial [91] and is comparable with the outcome of the ELIANA trial of tisa-cel with an overall survival rate of 55% (2022 EHA Congress, Abstracts #S112, #3782).

While the majority of trials test a single CAR there have been efforts to make direct clinical comparisons, albeit on a small scale, between CARs utilizing either CD28 or 4-1BB costimulatory domains. Cheng et al. infused seven patients with a 1:1 mixture of CAR-Ts produced with either FMC63-CD8α-CD28-CD28-3ζ or FMC63-CD8α-CD8α-41BB-3ζ CARs [33]. It is important to note that not only do the costimulatory domains differ, but so do the transmembrane regions, though the CD28 heterodimerization motif is absent as both CARs utilize a CD8α hinge. The authors transduced PBMCs with gamma retroviral vectors encoding either CAR separately, then combined the resulting products in a 1:1 ratio. They showed that the FMC63-CD8α-CD28-CD28-3ζ and mixed products produced more interferon gamma from the CD8 population and were more potent than the FMC63-CD8α-CD8α-41BB-3ζ product in a Raji mouse model. Following infusion with manufactured, mixed cells, five of seven patients achieved CR within one month, with one patient remaining in CR for 18 months, and a second patient in ongoing CR at 15 months at time of publication. In vivo expansion kinetics were monitored by qPCR and indicated that the FMC63-CD8α-CD28-CD28-3ζ cells peaked first around day 9, with the FMC63-CD8α-CD8α-41BB-3ζ cells peaking around day 13. These differences in kinetics are comparable to those seen with other 28-3ζ and 4-1BB-3ζ CARs in the clinic [17, 62–64]. There was no overall difference in expansion and persistence profiles for one CAR-T over the other, though this was a small sample size.

A more direct comparison was attempted by Li et al. [35]. In this study two CARs with IgG4 hinge, CD28 transmembrane and CD3ζ activation domains and either a CD28 costimulatory domain or 4-1BB costimulatory domain were compared in five patients each. While there was a higher peak of CAR-T cells in the 4-1BB group this was not statistically significant. In 9/10 patients CAR-T cells persisted for less than a month, probably contributing to all the patients, including the 7 complete responders, relapsing. The authors consider that their manufacturing process, based on OKT3 and IL2 activation, created a highly differentiated product that limited the potential for long term persistence. As such it is not possible to draw any meaningful comparison between the relative contributions of each costimulatory domain from this study.

Zhao et al. professed to comparing axi-cel and tisa-cel in both a preclinical and a clinical study [92] but this needs to be looked at with a degree of skepticism as the manufacturing method for the “axi-cel” material was based on lentiviral transduction of enriched T-cells, rather than gamma retroviral transduction of PBMCs. However, removing the variability in manufacturing method does allow a more direct comparison of the constructs on a similar background; it is important in a case like this to fully understand the question being asked, as in this case this is not a true comparison of the commercial products, rather it is focused on the CARs themselves. In this instance it was found that the tisa-cel-like CAR, containing the 4-1BB costimulatory domain was more potent and persistent in a mouse Nalm6 B-ALL model and in a small clinical trial with 36 r/r B-ALL patients (18 patients for each treatment). The authors note that their observations differ from earlier studies, notably that by Li et al. [35], but acknowledge that differences in architecture and manufacturing continue to make it difficult to compare across studies.

## Conclusions

CAR-T cell activity is dependent on a wide range of factors. While there are clear biological differences between CD28 and 4-1BB costimulation, these quickly become difficult to parse out from other variables such as the rest of the CAR architecture (including binder, hinge, and transmembrane domain) which can influence how the CAR interacts with not only the target antigen, but also other proteins on the T cell surface. Manufacturing methods, including starting material, vector, activation, and culture conditions can greatly influence the phenotype of the final product, which can impact potency and persistence. Finally, the differences in clinical trial design, including patient population, prior treatment, lymphodepletion methods, dose, and criteria for assessing response and adverse events, make it impossible to draw all but the broadest comparisons across different CARs to determine which costimulatory domain is more effective. In the future, the development of high throughput screening platforms coupled with predictive, biologically relevant assays will facilitate identifying optimal CAR architectures through a holistic consideration of the entire molecule.

## Data Availability

Not applicable.

## Abbreviations

APC: Antigen presenting cell
CAR: Chimeric antigen receptor
TCR: T-cell receptor
MHC: Major histocompatibility complex
ScFv: Single chain antibody variable region fragment
Ig: Immunoglobulin
CRS: Cytokine release syndrome
CR: Complete response
PBMC: Peripheral blood mononuclear cell
Axi-cel: Axicabtagene ciloleucel
Brexu-cel: Brexucabtagene autoleucel
Tisa-cel: Tisagenlecleucel
Liso-cel: Lisocabtagene maraleucel
